# Early Detection of Compartment Syndrome With Minimal Symptoms: A Case Report on Continuous Pressure Monitoring

**DOI:** 10.7759/cureus.74453

**Published:** 2024-11-25

**Authors:** Abdulrhman M Al Nasser, Edward J Harvey, Alexandra C Bunting

**Affiliations:** 1 Surgery, McGill University, Montreal, CAN

**Keywords:** compartment syndrome, pressure, sensors, surgery, trauma

## Abstract

Compartment syndrome (CS) arises from various etiologies but is most commonly associated with severe traumatic injuries. It is a difficult diagnosis to make in a timely fashion because clinical signs and symptoms are subjective. Missing the diagnosis is a devastating mistake for the patient and the physician. There has been protracted debate over the effectiveness of clinical signs and symptoms, particularly concerns over their sensitivity and specificity. Both missed diagnoses and unneeded prophylactic releases are costly to the health system. A desired device would be an objective tool that decreased false positives and negatives while ensuring diagnosis in a timely fashion of true positives. The treatment for CS is immediate fasciotomy, but fasciotomy is not a complication-free procedure. Physicians need to be sure of the diagnosis both in order not to have the devastating consequence of a missed case but also not to perform with prophylactic fasciotomies that add to patient complications and the cost of treatment. Previous care maps usually resulted in fasciotomy being performed in extremities that will not or have not yet developed CS. New technology that allows monitoring of continuous pressure monitoring seems to currently be the best aid to diagnosis. We present our experience in using continuous pressure monitoring in decreasing time to diagnosis in a case post-trauma of a lower limb with minimal pain.

## Introduction

Compartment syndrome (CS) is a limb-threatening condition. The diagnosis of CS, as classically taught, still currently relies on clinical findings of increasing pain, paresthesia, and paralysis. Unfortunately, the clinical signs are subjective. Pressure has been used to aid in diagnosis by some investigators with mixed results. Early methods used for measuring intracompartmental pressure (ICP) had limited accuracy [1,2]. Missed diagnoses result in large muscle deficits, flap revision surgeries, kidney damage, amputation, and even death [3,4]. Duckworth et al. began a program of repeated pressure measurement at their center [3]. Monitoring continuous pressure was shown to decrease the time to surgery and aid in diagnosis. Gold standard treatment of CS consists of emergency fasciotomy to reduce the pressure within the compartment [4]. The surgical wound often must be left open for an extended period, and the necessity for delayed closure of the wound carries a high risk of infection [4-6]. An in-depth analysis of current databases shows late diagnosis has increased complications of muscled death while still overdiagnosing CS and resulting in early prophylactic surgery [7]. Big data analyses have shown that almost one in six fasciotomies of the injuries of the leg revealed some degree of myonecrosis and 5.4% of fasciotomies led to amputations [7]. This indicates that current techniques used for the diagnosis of CS may be inadequate [7], resulting in late treatment or unnecessary interventions. This is illustrated by the fact that a missed diagnosis of CS represents a large medicolegal risk. This risk changes the way that surgeons think of the disease and changes practice to a more legal preventative course [8,9]. The use of a new micro-electrical machine system (MEMS) based pressure monitor [2] has shown good results in trauma patients [10-12]. MEMS allows the insertion of the pressure sensor and computing chip into the area of concern. Direct measurement without a column of water is much more accurate (600%) and does not require an expert in the management of the sensor and fluid column [2]. This has been seen in a quicker time to surgery, avoidance of any late CS cases, and a decrease in the number of fasciotomies - not just in the prospective cohort trials but in large clinical releases. Further publications of the larger clinical experience will better define usage.

## Case presentation

**Figure 1:**
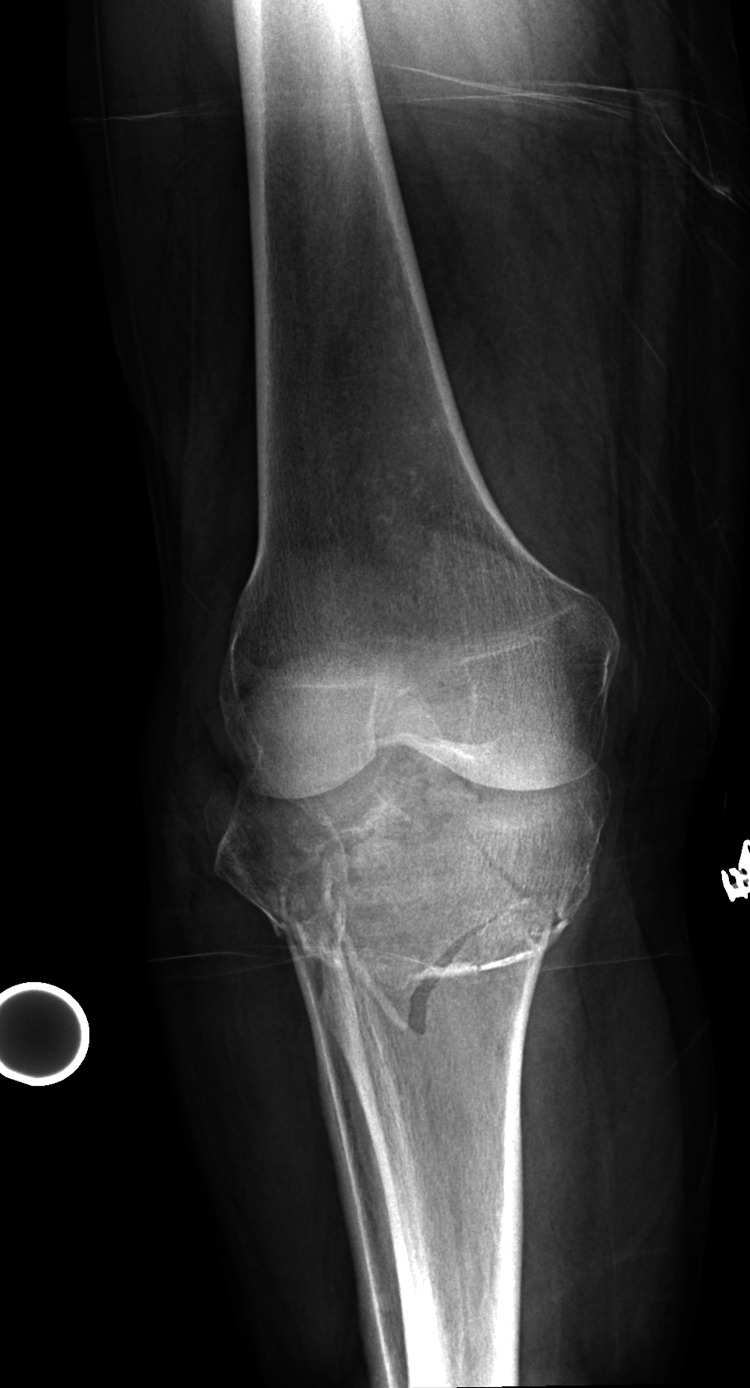
AP radiograph of the injured limb. The fracture is a comminuted but impacted proximal tibia fracture.

**Figure 2:**
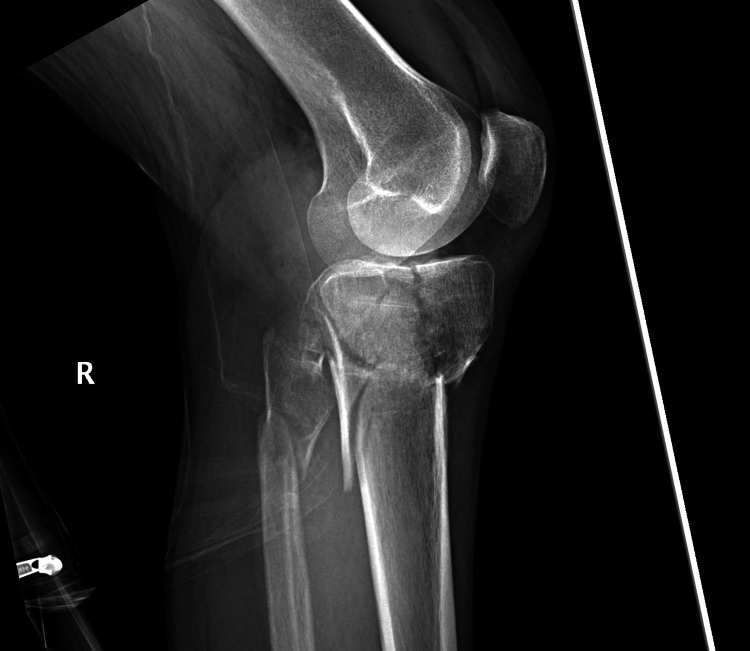
Lateral radiograph of the injured limb. There is minimal displacement on the lateral view of the knee. Mildly displaced fibula fracture is also visible.

The patient was a 53-year-old female, a previously healthy patient, who presented to the orthopedic team in the emergency department 12 hours after being struck by a truck while riding her bike. She had an episode of loss of consciousness at the time of the injury. Her neurological examination and head scan were negative. The patient was able to easily move her right leg without pain. The patient was not complaining of excruciating pain sometimes seen in CS. Classically pain to passive motion is seen and was not exhibited here. Pain to direct palpation over the right knee resulted in the performance of a knee and tibia radiograph (Figures 1-2).

**Figure 3:**
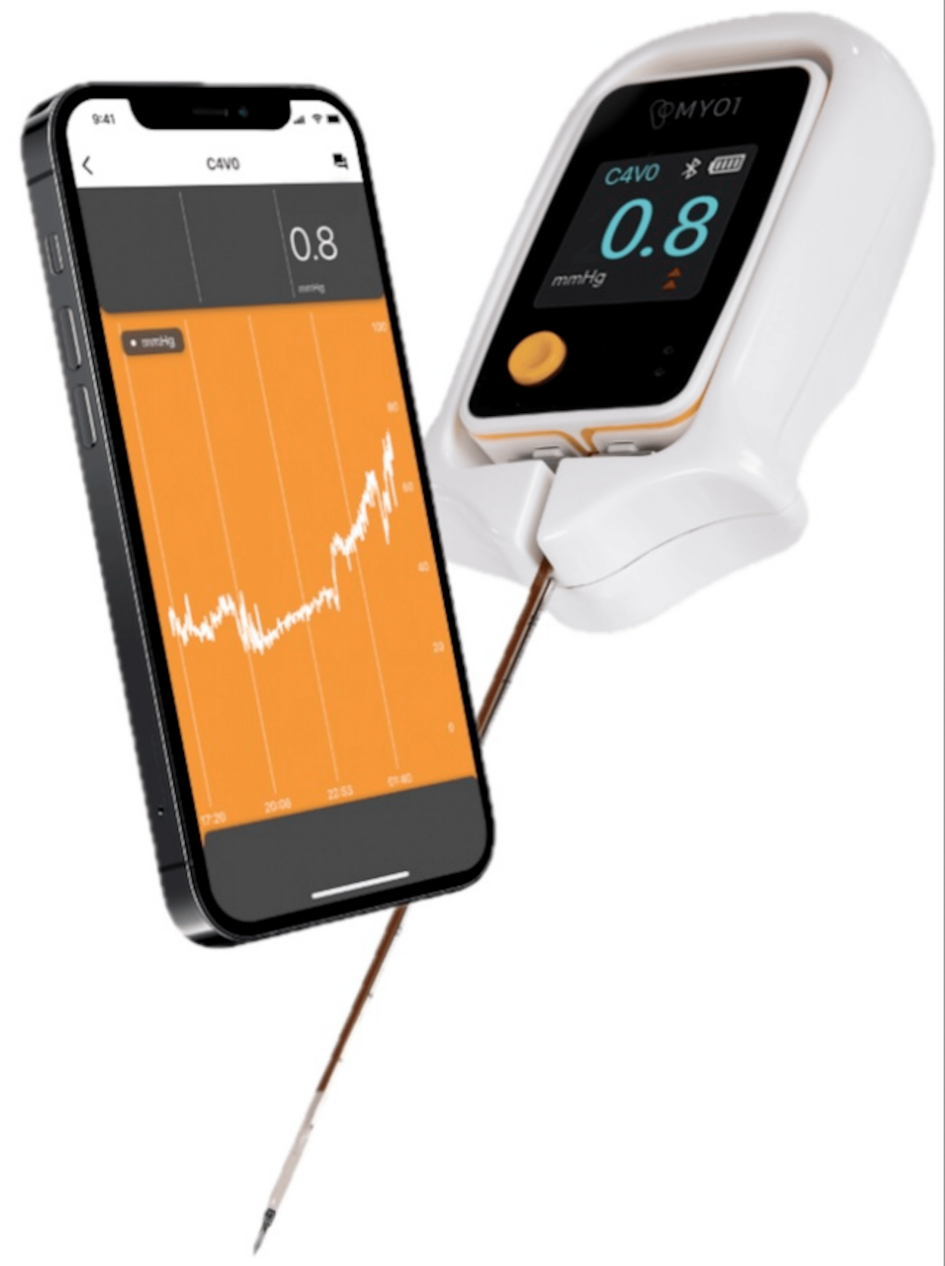
MEMS-based continuous pressure monitor. This is a single-use sterile medical device designed to be placed in the muscle belly of the most affected compartment. MEMS: micro-electrical machine system Figure supplied by MY01 Inc., Montreal, Canada

**Figure 4:**
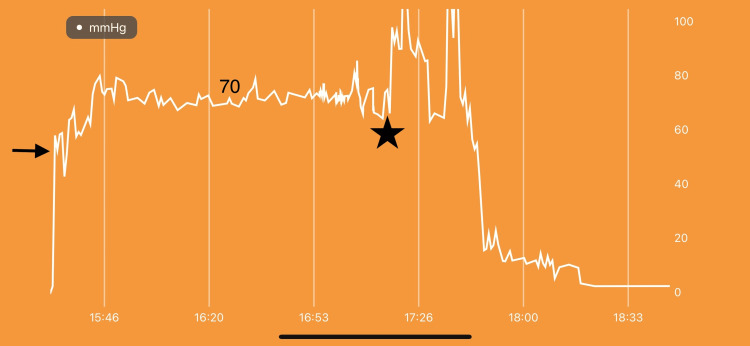
Pressure measurement over the initial period of monitoring until surgery. X-axis is time. The y-axis is pressure in mmHg. The arrow shows the first pressure measurement after the insertion in the anterior compartment. Number 70 shows pressure during most of the observation period. The star is the point where pressure increased above 100 mmHg just before surgery.

Examination of the right leg revealed normal color (no pallor) and normal temperature (no poikilothermia). Pulses were equal bilaterally with good tissue oxygenation in the right foot. There were no paresthesias. She had some mild pain (3/10) in the right knee but no increase in pain with passive motion. By clinical exams, she did not have CS. The team was debating discharge home and surgery later in the week. Because of an internal care protocol pertaining to proximal and midshaft tibial fractures, it was opted to add continuous ICP monitoring in order to aid clinical examination. The MEMS-based pressure monitor [2] (MY01 Inc., Montreal, Canada; Figure 3) was inserted as per protocol in the anterior compartment of the right leg. This device produces a phone app-based graph of time versus pressure. There is also an output display on the leg itself for direct observation of ICP [13-16]. Preclinical and clinical studies have shown that this is more accurate than previous technology [10]. MEMS-based sensors are a modern manufacturing technology that allows the placement of a silicon-based sensor directly into the muscle belly for a more accurate pressure value [2]. It also allows continuous real-time pressure monitoring. The literature around this new methodology has validated new standards in CS diagnosis and treatment [10]. While the MEMS sensor was being inserted, the patient’s main complaint was bladder pain and shivering - not related to her fracture. The initial ICP measured upon insertion was 55 mmHg. This increased to 70 mmHg over the next 30 minutes (Figure 4). Pressure remained at 70 mmHg with a delta P of 10 for the next two hours.

**Figure 5:**
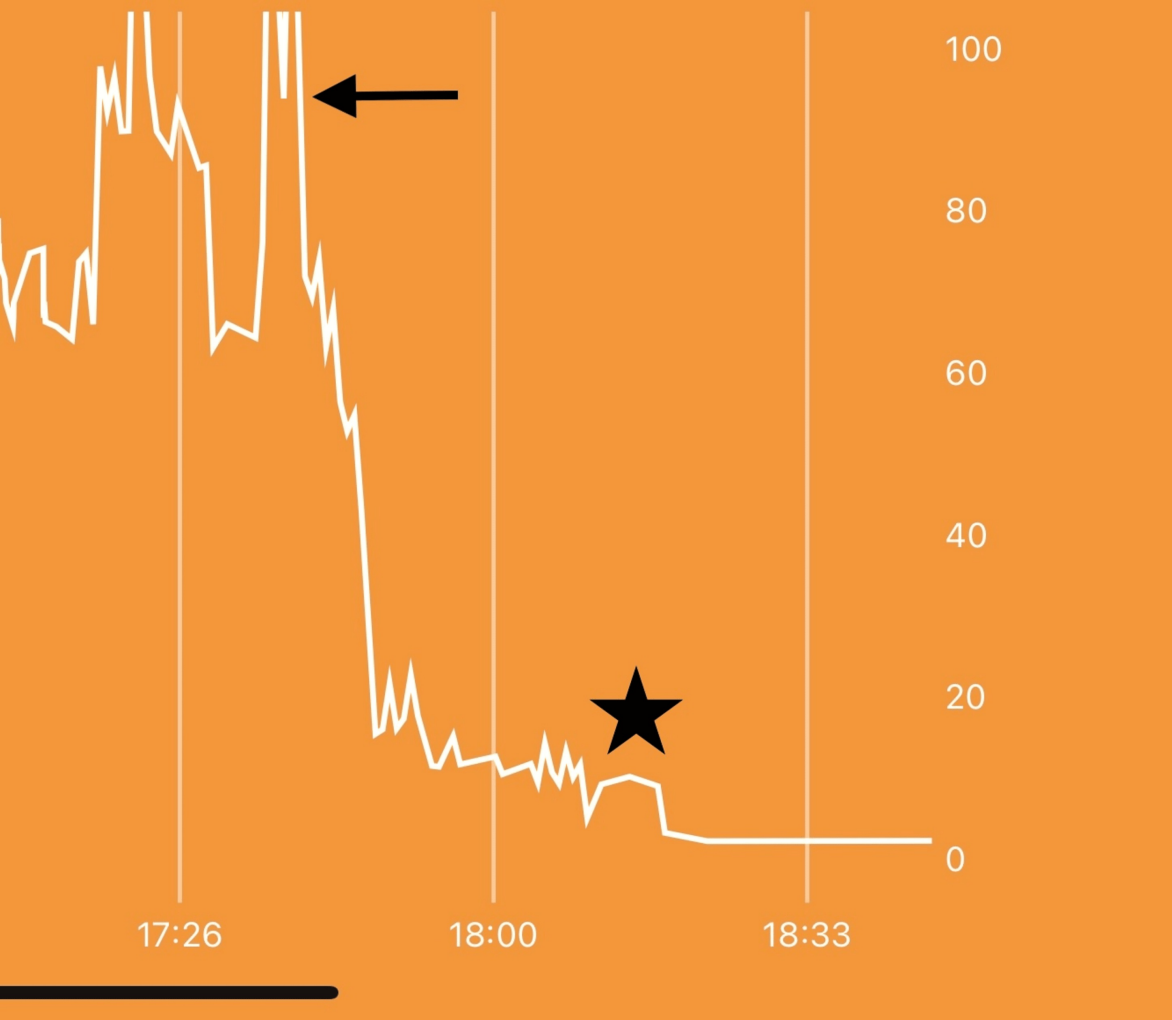
Pressure measurement during and after surgery. X-axis is time. The y-axis is pressure in mmHg. The arrow shows the time of release in the operating room. The star represents the pressure curve after two fasciotomy releases and transfer to the recovery room.

The patient was prepared for surgery based only on pressure trends as it was felt that she would develop CS despite the physical exam and lack of symptoms. It was not until the patient was brought into the operating room that her pain in the leg increased. At this time, the patient complained of severe tenderness over the ankle and dorsum of the foot while still only having mild pain with passive toe and ankle motion. The compartments were compressible without increased pain. Because of the protracted low delta P, it was opted to perform fasciotomy plus external spanning fixation on the patient. During induction, the patient had more pain in the leg around the knee. The ICP increased to over 100 with a negative delta P. It was elected at this time to perform four-compartment fasciotomies as it was felt that there was an impending CS. All compartments were released in the normal fashion with full-length fasciotomies. A two-incision release was carried out with the resultant bulging dusky muscle that returned to a bright red color over the course of the procedure. The device was allowed to remain during surgery, and the complete release of the compartments can be observed on the app tracing (Figure 5). The patient was treated with spanning external fixation and fasciotomy with definitive open reduction and internal fixation at follow-up surgery.

Vacuum-assisted closure was used over the next three days. Closure of the skin was accomplished at the next surgery. The lateral incision was difficult to close, needing skin stretching and advanced techniques. This was a grade 2 CS - a true positive early case with bulging of the musculature such that primary closure was impossible [17]. This classification system runs from grade 1 - prophylactic in a leg without CS to grade 5, an amputation from CS. This case would presumably have resulted in a higher-grade injury without early intervention as the pressures increased. If the diagnosis was not facilitated by pressure measurement, there could have been a worse outcome. Early detection avoids permanent muscle death [18] as evidenced in the trials of pressure monitoring from McQueen et al. and Leighton et al. [10,18].

## Discussion

There was no strong clinical indication that this patient had CS. This illustrates, at least, in this case, the weakness of relying on the classic Ps for diagnosis. Clinical signs were not enough for this case. All Ps have been shown to be not specific and sensitive. Implementation of an institutional protocol for monitoring and the use of continuous pressure monitoring allowed early diagnosis before permanent muscle damage occurred. The institutional policy on the implementation of muscle continuous pressure monitoring includes proximal and mid-shaft tibia fractures (OTA Class 41, 42B, and 42C). Both ICP trend and delta P are seen as important factors in predicting CS. Decreasing the time to diagnosis means decreased muscle death [18] as per McQueen et al. McQueen et al. advocated for decompression based primarily on pressure measurements, arguing that this approach significantly reduces the time to definitive treatment and improves outcomes by preventing missed diagnoses. They, in particular, viewed trends in delta P over time as the key to diagnosis. Clinical findings have poor sensitivities (13-64%) compared to ICP monitoring [13,18]. McQueen et al. went so far as to say that decompression be carried out primarily on the basis of pressure measurement as this results in a reduced time to definitive treatment when compared to waiting for the development of clinical symptoms and signs. This allows the physician to obtain more early-grade cases and avoids missed diagnoses where the patient has poor outcomes [10,17,18].

Usage of the MEMS device has been effective in decreasing complications [10]. There are perceived and real limitations in introducing new technology. Concerns over cost-effectiveness were examined by the York Health Economics Consortium (YHEC) in preparation for European release. The YHEC group felt that at a price point of up to 4,000 dollars a device, with sensitivity and specificity of over 94%, would save money for the healthcare institute [10]. Accessibility concerns are real for any new paradigm-shifting technology, but the device is FDA and CE mark cleared for regular sales. The learning curve has not been steep as the device requires placement anywhere a muscle belly of concern, so it is easier to insert than even an intravenous line.

The important innovation in care with this method of monitoring MEMS-based continuous pressure measurement is early recognition of true positives. There has been a reduction in time to OR and unneeded fasciotomies with this technique of monitoring [9]. Earlier work by McQueen et al. [18], amongst others, is validated with recent clinical data [10] on continuous compartment pressure monitoring. We now know from recent literature and cases like this one that clinical signs are not reliable [13] and that older techniques for pressure monitoring are inaccurate [1,2,13]. Newer technology allows better aid in diagnosing CS, particularly for these patients.

## Conclusions

MEMS-based continuous compartment pressure measurement represents a new significant aid in the diagnosis of CS. Usage in preclinical and clinical traumatic cases has revealed high specificity and sensitivity, allowing early diagnosis and avoidance of false positives. This innovative device allowed early diagnosis of CS in this trauma case with no clinical signs. Continuous pressure measurement will play an important role in the diagnosis of true positive cases while decreasing the number of unnecessary surgeries (false positives and prophylactic surgeries). This represents a new method of controlling patient morbidity and minimizing hospital costs as well as ensuring maximal patient outcomes.
